# Concept of Similarity Method for Prediction of Fatigue Life of Pavement Structures with HiMA Binder in Asphalt Layers

**DOI:** 10.3390/ma14030480

**Published:** 2021-01-20

**Authors:** Magdalena Złotowska, Roman Nagórski, Krzysztof Błażejowski

**Affiliations:** 1 Institute of Roads and Bridges, Faculty of Civil Engineering, Warsaw University of Technology, al. Armii Ludowej 16, 00-637 Warsaw, Poland; r.nagorski@il.pw.edu.pl; 2 ORLEN Asfalt sp. z o.o., ul. Chemików 7, 09-411 Płock, Poland; krzysztof.blazejowski@orlen.pl

**Keywords:** flexible road pavement, highly modified asphalt binder, HiMA, estimation of pavement life, similarity method

## Abstract

Since 2009, SBS (styrene-butadiene-styrene) polymer-modified binders (HiMA—Highly Modified Asphalt) have been tested worldwide. Highly modified binders are characterized by extraordinary properties resulting from the reversal of the binder-polymer phase. This contributes to very good test results for asphalt mixes. The use of such a modern binder poses a challenge in terms of structure design, mainly due to the lack of a recognized and calibrated method suitable for this material. The article proposes a new approach to pavement fatigue life estimation—the Similarity Method—which is based on the use of AASHTO 2004 equations and laboratory fatigue testing results of asphalt concrete mixes for asphalt base course. The article presents the method and the results of its sensitivity testing with respect to the influence of the material (type of asphalt concrete), thickness of the asphalt base course, stiffness of the subgrade and the assumed *FC* index (the area of bottom-up cracks). The results of fatigue life according to the Similarity Method are within the range of values obtained for AASHTO 2004 and the fatigue results according to the equations obtained in the laboratory. This approach will enable inclusion of new materials, such as HiMA asphalt mixtures, in pavement structure design.

## 1. Introduction

### 1.1. Background

Development of highly polymer-modified binders (HiMA, HMB, HPMB, further referred to as HiMA—Highly Modified Asphalt), especially those modified with elastomers [1,2,3,4,5,6,7,8], not only improves the potential for construction of a high-performance pavement without increasing the thickness of asphalt layers, but also offers the possibility of reducing the thickness while maintaining the design life of the pavement [9]—as compared to the previous practice using unmodified and modified binders [10]. Highly elastomer-modified binders are a material with high SBS (styrene-butadiene-styrene) block copolymer content, usually above 7.0% *m*/*m*, which, in consequence of the bitumen modification process, leads to phase reversal in the material—the SBS phase dominates over the bituminous base phase. The resulting highly modified binder exhibits some of the elastomeric characteristics to a greater degree, while the influence of the characteristics associated with bitumen in the final material is reduced. In particular, the HiMA type binders exhibit high elastic return properties (more than 90% according to EN 13398 [11]) and reduced stiffness at intermediate and low temperatures. Consequently, asphalt mixtures with highly modified binders have many advantages in terms of physical and mechanical properties. They are more flexible, with higher strain tolerance, and more resistant to cracking than unmodified asphalt mixes [12]. Therefore, with a lower stiffness modulus, HiMA asphalt mixes are extremely resistant to fatigue cracks despite the increased strain. Similarly, at low temperatures, due to the lower stiffness of the binder, cracking occurs later than in mixtures with standard (unmodified) binders. This has been proven by the results of fatigue testing of asphalt mixtures using the 4PB-PR, TSRST and SCB methods [13,14], but also by the results obtained in the bending beam rheometer BBR [15] and in the LAS test [16,17].

### 1.2. Basis of Research

Despite the favorable features of highly modified asphalts [18], which were recognized over a decade ago, taking into account the impact of their parameters on the design life of the structure still proves challenging. Although the results of laboratory fatigue tests of asphalt mixtures with HiMA binders indicate durability that is many times greater than in the case of unmodified asphalt mixtures [2,19], the fact that the standard formulas used to calculate the fatigue life of the pavement do not enable direct evaluation of the benefits of using highly modified binders with SBS polymers (lower layer stiffness and higher critical structure strain vs. higher fatigue resistance of the asphalt mixture) is still problematic. Determination of the fatigue life of pavement layer systems that include HiMA asphalt layers poses significant difficulties due to the fact that the literature still lacks sufficiently accurate, reliable and relatively simple tools to quantify the fatigue life and, more broadly, the durability of such pavements [20].

Several attitudes have been proposed to address this problem. Firstly, the results of “laboratory fatigue life” of the HiMA asphalt mix used in the lower asphalt layer of the pavement can be transferred into the predicted life of the entire pavement structure [21] using a transfer function. Unfortunately, no relatively simple mathematical formulas for this transfer have been obtained yet [20,22]. A complex algorithm for transferring “laboratory fatigue resistance” (expressed in ε_6_—value of strain corresponding to fatigue life equal to *N*_f_ = 10^6^ cycles of loading, and S_mix_—dynamic stiffness modulus of the asphalt mix for a specific frequency, e.g., 10 Hz) to “structural fatigue resistance” can be found in the Austrian method [23]. Likewise, HiMA mixture fatigue curves may be used to determine Fatigue Endurance Limit (FEL) [24,25,26,27,28,29,30]. Secondly, creation of formulas for calculation of the predicted pavement life is possible by adapting the formulas found in the M-EPDG AASHTO 2004 method [31,32], the Asphalt Institute method [33], the Shell method [34], the French method [35] as well as in many American works—and, in particular, in the Czech method [36], which is close to this objective)—by creating empirical calibration coefficients for pavements that include the HiMA binder. Thirdly, applying the mechanistic approach is possible by treating the development of pavement degradation as a weakening phase, taking it into account in the constitutive equations and determining the life of the structure on this basis; an example of such an approach, including viscoelastic effects, is the use of the VECD theory and its S-VECD application in the FlexPave program [37].

The major shortcoming of the laboratory methods of fatigue life determination is the mismatch between the criterion of failure adopted in these methods and the criterion of fatigue failure of the entire pavement, expressed as the degree of its surface distress [38]. This leads to significant differences in the predicted “laboratory fatigue life” compared to “structural fatigue life”. On the other hand, the formulas for forecasting of the “structural life” should be calibrated (in situ) for a specific geometric and material design of the pavement. For standard flexible pavements with asphalt layers, this has been accomplished in several centers, including the Asphalt Institute [33], AASHTO [31,39], Shell research center [34], LCPC [35] and others. In the case of highly polymer-modified binders, such work is in progress [40], but the results, once obtained, will still remain at the verification stage for a considerable time. Nonetheless, application of a strictly mechanistic approach to description of structure degradation, in particular to pavement life prediction, remains essentially in the area of scientific research without wider use in practice.

### 1.3. Objective

The paper presents the use of HiMA materials in the pavement structure design. The aim of this work is to present the authors’ proposal of a method for prediction of design life values due to fatigue cracking of pavements that include asphalt layers with highly modified binders. This method, called the Similarity Method (SiM), is based on a certain realistic conversion of the pavement lifespan calculated on the basis of laboratory fatigue test of an asphalt mixture. It is grounded on a hypothesis assuming that the laboratory fatigue equation of an asphalt mixture with HiMA has to be related to such an equation of the layer (set of layers) of the structure with this mixture in the asphalt base course. The relation should be analogous to the manner in which the laboratory-determined fatigue equation of a mixture with unmodified binder is related to the fatigue equation of pavement structure with this mixture in the asphalt base course, e.g., according to the AASHTO 2004 formula.

### 1.4. Scope of the Research Program

The general presentation of the Similarity Method is illustrated for a typical flexible pavement structure, for two material variants of the asphalt base course—asphalt concrete with neat binder and with highly modified HiMA binder. The remaining layers and subgrade were identical in both analyzed structures. Additionally, sensitivity of the method was tested by comparing the results of the calculated predictions of pavement fatigue life for four other cases: a change of asphalt concrete grading in the base course (material sensitivity), a change in the thickness of the asphalt base course (geometric sensitivity), an increase in the modulus of the improved subgrade (sensitivity to stiffness of the improved subgrade), and for a different value of the “bottom-up” mesh cracking index *FC* on the road surface (sensitivity to the assumed index defining the fatigue failure of the pavement).

Asphalt concrete mixtures for the base course were tested at 10 °C/10 Hz in the 4PB-PR fatigue test—four-point bending of prismatic beams according to EN 12697-24 [41], obtaining fatigue life characteristics. The master curves at this temperature were also determined (complex moduli of the mixtures for the normal load cycle frequency sequence). The values of the Huet-Sayegh model parameters for the mixtures were determined and verified. The values of dynamic stiffness moduli determined on the basis of the Huet-Sayegh model are consistent with the values determined from laboratory tests, which were used in the fatigue cracking resistance prediction formulas.

The critical strains in the analyzed layer systems were calculated using the VEROAD program [42], using the Huet-Sayegh model [43] for asphalt layers at an equivalent temperature of 10 °C and at a typical speed of heavy vehicles (10 Hz load frequency).

The design life predictions due to fatigue “bottom-up” cracking expressed as the number of passes of standard 100 kN axles were determined based on:

1. the Similarity Method (the obtained value hereinafter referred to as “SiM fatigue life”, marked as *N*_f(SiM)_),
2. empirical formulas of the AASHTO 2004 method (the obtained value hereinafter referred to as “structural fatigue life”, marked *N*_f(struct)_) [31],
3. fatigue equations determined in laboratory (the obtained value hereinafter referred to as “laboratory fatigue life”, denoted *N*_f(lab)_) [17].

The paper does not encompass the issues of top-down cracking of asphalt layers [44,45], resistance to permanent deformation of asphalt layers (mix rutting) [46], design life of geogrid-reinforced asphalt pavements [47] and aging of asphalt layers.

## 2. The Similarity Method

The authors of the paper formulated a hypothesis that presents a formula for estimating the fatigue life of a pavement structure due to “bottom-up” fatigue cracks.

The key premise of the proposed method is the form of each formula used for calculating fatigue life according to the Wöhler’s concept:

(1) $$N_{f}=C{\left(\frac{1}{\varepsilon_{\mathrm{cr}}}\right)}^{\alpha },\;\mathrm{therefore}\;\log\left(N_{f}\right)=\log\left(C\right)-\alpha \log\left(\varepsilon_{\mathrm{cr}}\right)=A-\alpha \log\left(\varepsilon_{\mathrm{cr}}\right)$$

where ε_cr_ is the critical strain, on which the design life of the structure or pavement layer *N*_f_ mainly depends, and *C* and *α* are often products of coefficients dependent on specific factors and are subject to experimental calibration. Let us assume two different flexible structures S1 and S2, such that:

- The S1 (reference) structure contains asphalt layers only with such road asphalt for which the correct *N*_f_ fatigue life formula for the S1 structure, i.e., *C* = *C*_struct_ and α = α_struct_ are known and widely accepted—for example, the specific formulas of AASHTO, Asphalt Institute, Shell, etc. (cf. the RS structure presented in Section 3.1).
- The S2 (analyzed) pavement structure differs from the S1 structure only by one factor, i.e.,
  - the binder type in the lowest asphalt layer or
  - the binder type in all asphalt layers
  (for example, highly modified asphalt HiMA used instead of unmodified binder).
- Fatigue curves, i.e., *A* = *A*_lab_ and *α = α*_lab_ coefficients for the asphalt mixtures used in the asphalt base course of the S1 and S2 structures have been determined in the laboratory.

In such a case the formulated hypothesis states that the coefficients A and α for both structures S1 and S2 and for both mixtures in the zone of fatigue crack initiation (in the asphalt base course) are proportional, i.e.,

(2) $$\frac{A_{\mathrm{struct}}^{S2}}{A_{\mathrm{struct}}^{S1}}=\frac{A_{\mathrm{lab}}^{S2}}{A_{\mathrm{lab}}^{S1}},\;\frac{\alpha_{\mathrm{struct}}^{S2}}{\alpha_{\mathrm{struct}}^{S1}}=\frac{\alpha_{\mathrm{lab}}^{S2}}{\alpha_{\mathrm{lab}}^{S1}},$$

in other words, for both S1 and S2 designs the ratios of the structural coefficients A and α are identical to the respective ratios of A and α obtained in the laboratory for the mixture in which fatigue cracks are initiated.

The obtained equations

(3) $$C_{\mathrm{struct}}^{S2}=10^{A_{\mathrm{struct}}^{S2}},\;A_{\mathrm{strcut}}^{S2}=\frac{A_{\mathrm{lab}}^{S2}}{A_{\mathrm{lab}}^{S1}}A_{\mathrm{struct}}^{S1},\;\alpha_{\mathrm{struct}}^{S2}=\frac{\alpha_{\mathrm{lab}}^{S2}}{\alpha_{\mathrm{lab}}^{S1}}\alpha_{\mathrm{struct}}^{S1},\;A_{\mathrm{struct}}^{S1}=\mathrm{Log}\;C_{\mathrm{struct}}^{S1},$$

enable the following estimation:

(4) $$N_{f\left(\mathrm{SiM}\right)}=N_{f\left(\mathrm{struct}\right)}^{S2}=C_{\mathrm{struct}}^{S2}{\left(\frac{1}{\varepsilon_{h}}\right)}^{\alpha },\;\alpha =\alpha_{\mathrm{struct}}^{S2},$$

The above algorithm will be applied to the analyzed example structures described in Section 3.

## 3. Calculation Data

### 3.1. Pavement Structure

For the analysis, a typical layer system of flexible pavement structure dedicated for total traffic in the range of 7.4–22.0 million standard 100 kN axles was adopted in two variants:

- RS structure (reference)—typical flexible structure used in Poland, with unmodified road binder 35/50 ([48], pen@25 °C = 35–50) in the asphalt base course made of AC 22 asphalt concrete [49],
- HB structure (HiMA base)—structure using highly modified asphalt HiMA binder in the AC 22 base course [49], other material parameters as in the RS structure.

**Figure 1:**
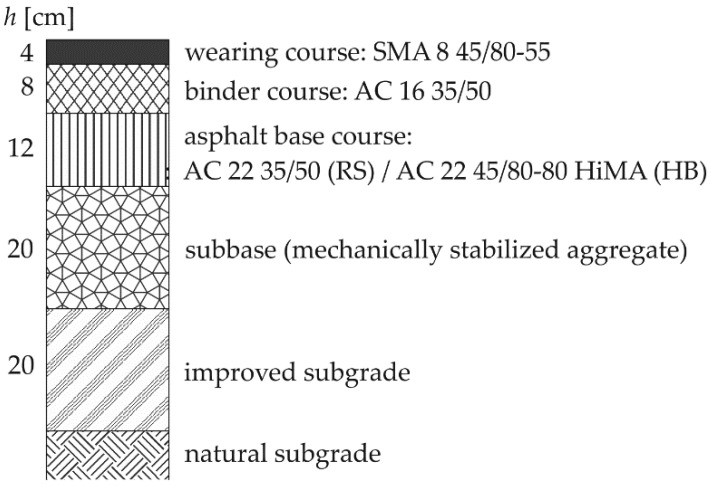
Pavement structure for analysis.

Figure 1 shows the layout of the layers of the analyzed structures with the type of asphalt mix and the type of binder used.

All asphalt mixtures were designed on the basis of Polish requirements for national roads, according to the document WT-2 2014 [50] developed by the central road administration (GDDKiA). Each type of mix, regardless of the type of binder, was characterized by the same NMAS and gradation for all variants. The characteristics of the highly modified PMB 45/80-80 HiMA binders were in accordance with the Polish national appendix to EN 14,023 [51], edition 2014 [52]. The essential parameters are given in the Table 1.

According to the proposed concept of the Similarity Method, the RS structure was assumed to be S1 and the HB structure to be S2.

### 3.2. Mechanical Model of Pavement and Materials. Material Parameters

For modeling of the analyzed pavements, a linear model of layered material half-space was used as a general initial mechanical model. It consisted of unlimited (in horizontal directions) homogeneous and isotropic layers of constant thickness, representing structural layers and improved subgrade, and a homogeneous and isotropic half-space representing natural subgrade [53]. For comparative analytical purposes, full continuity of displacements on layer interfaces was assumed, meaning full bonding of pavement layers.

The viscoelastic Huet-Sayeh model (hereinafter abbreviated to H-S) [53,54] was used to describe the material properties of asphalt pavement layers. For the remaining (non-asphaltic) layers, Hooke’s elastic model (hereinafter abbreviated to H) [53] was used. Accordingly, the constitutive equations of these materials in the “stress-strain” relation in the case of the H-S model may be given in the following (operational) form:

(5) $$\sigma =E_{H-S^{\epsilon }},\;E_{H-S}=E_{p}+{\left[\frac{1}{E_{a}}+\frac{1}{E_{a}^{*}}+\frac{1}{E_{b}^{*}}\right]}^{-1}=E_{p}+\frac{E_{a}}{1+\delta {\left(\tau^{k_{a}}D^{k_{a}}\right)}^{-1}+{\left(\tau^{k_{b}}D^{h_{b}}\right)}^{-1}}$$

(6) $$E_{a}^{*}=\frac{\eta_{a}}{\tau }\tau^{k_{a}}D^{k_{a}},\;E_{b}^{*}=\frac{\eta_{b}}{\tau }\tau^{k_{b}}D^{h_{b}},\;\delta =\frac{\eta_{b}}{\eta_{a}},\;\tau =\frac{\eta_{b}}{\eta_{a}},\;D^{\alpha }=\frac{d^{\alpha }}{dt^{\alpha }},$$

where *E*_a_, *E*_p_, *η*_a_, *η*_b_, *k*_a_, *h*_b_ are the parameters of the model, $D^{\alpha }$ means an operation of differentiation of the order α (rational number). In the case of the H model the relation is expressed by:

(7) σ=Eε,

where *E* is the modulus of elasticity.

The values of material parameters of the H-S model of asphalt layers were assumed for the equivalent temperature of T = 10 °C (for the entire year) and for frequency *f* = 10 Hz (corresponding to the typical speed of heavy vehicle traffic, estimated as 60–75 km/h under free flow traffic conditions). These values, determined from the master curves on the basis of measurements of complex stiffness moduli E* for a specific set of frequencies *f* from the four-point bending of samples according to the standard [41], were taken from the work [19].

In relation to asphalt layers, the dynamic stiffness moduli |E*| (E* is the complex stiffness modulus) used to predict the fatigue life of the pavement, were also given (determined experimentally with the material parameters of H-S models).

The values of Young’s *E* modulus of elasticity of materials in non-asphaltic layers and the values of Poisson’s *ν* coefficients of materials of all pavement layers were adopted in accordance with the Polish technical requirements [55].

The values of material parameters of pavement layers in the adopted H-S and H models are listed in Table 2, Table 3 and Table 4. Table 3 shows the values of *V*_a_ and *V*_v_ parameters of the mixtures used in the considered structures, obtained on the basis of measurements [19].

### 3.3. Pavement Load

The load of the pavement is a vertical force *P* = 50 kN, representing a standard single vehicle wheel with the value resulting from the design (equivalent) axle load of 100 kN, distributed evenly with the intensity *p* on a circular surface of radius *a*, where *p* = 850 kPa is assumed and, consequently, *a* = 13.68 cm. This load is movable along the direction of the road axis, with a constant speed of *υ* = 60 km/h.

### 3.4. Design Life Based on AASHTO 2004

The design life, determined by the AASHTO 2004 method for the entire cross-section of the pavement structure—with fatigue cracks limited to several percent of the total lane area in the case of “bottom-up” cracks—is expressed by the following formulas:

(8) $$N_{f\left(\mathrm{atruct}\right)}=C_{s}{\left(\frac{1}{\varepsilon_{h}}\right)}^{\alpha },\;C_{s}=D_{\mathrm{FC}}C_{\mathrm{ph}}C_{m},\;\alpha =3.9492,$$

where

(9) $$C_{\mathrm{ph}}=7.3557\cdot 10^{M},\;M=4.84\times \left(\frac{V_{a}}{V_{a}+V_{v}}-0.69\right),\;C_{m}=k_{1}\left(10^{-6}\right){\left(\frac{1}{S_{a}}\right)}^{1.281},\;k_{1}=\frac{1}{0.01+\frac{12}{1+e^{\left(15.676-1.1097\cdot h_{\mathrm{ac}}\right)}}},\;D_{FC}=\frac{1}{100}10\;\left[-C_{1}C_{1}^{\prime }+\ln\left(\frac{100}{FC}-1\right)\right]\;\frac{1}{C_{2}C_{2}^{\prime }},\;C_{1}=1.0,\;C_{1}^{\prime }=-2C_{2}^{\prime },\;C_{2}=1.0,\;C_{2}^{\prime }=-2.40874-39.748{\left(1+\frac{h_{\mathrm{ac}}}{2.54}\right)}^{-2.856}$$

and:

*N*_f_—fatigue life [number of standard axles], *S*_a_—stiffness modulus of the lowest asphalt layer in which cracks are initiated (assumed to be equal to |E*|) [MPa], *ε*_h_—maximum tensile strain (in the horizontal direction) on the bottom of the asphalt layer in which cracks are initiated, *V*_a_—binder content by volume in the mix used in this layer [% *v*/*v*], *V*_v_—air void content by volume in the mix in this layer [% *v*/*v*], *k*_1_—parameter specified in the formula (1) calibration process, depending on the total thickness of asphalt layers in the case of “bottom-up” cracks, *h*_ac_—total thickness of asphalt layers [cm], *D_FC_*—total fatigue damage to the pavement in the case of fatigue cracks occurring on the *FC* percentage of the total lane area, C_1_, C_1_′, C_2_, C_2_′—calibration coefficients.

The calculation assumes *FC* = 5%.

### 3.5. Laboratory Fatigue Life

Laboratory fatigue equations were also determined for the tested asphalt mixtures. Such an equation has the form (1):

(10) $$\log N_{f}=A_{1}\log\left(\varepsilon_{t}\right)+A_{0}$$

where *ε*_t_—amplitude of cyclic tensile strain of the specimen, applied with a frequency of *f* = 10 Hz in the four-point bending test (4PB-PR) at *T* = 10 °C, according to the standard [41], for which *N*_f_ represents the conventional fatigue life of the mixture, i.e., the number of strain cycles at which the initial value of the dynamic stiffness modulus |E*| of the mixture is reduced by half.

The fatigue test was performed at four strain levels: 150, 200, 250 and 300 με for the AC 22 35/50 asphalt mixture and 300, 350, 400 and 500 με for the AC 22 PMB 45/80-80 HiMA mixture. At least 3 samples were tested at each strain level. The values of the coefficients *A*_0_ and *A*_1_ determined on the basis of the tests and given in the work [19] are presented in Table 5.

Values of fatigue lifespan *N*_f(lab)_ determined from fatigue curves (10) for strains of *ε*_t_ = *ε*_h_ calculated for real structures are incomparable directly with the design life *N*_f(struct)_ determined on the basis of AASHTO formulas (8)–(9) which pertain to the behavior of the entire pavement structure and a different failure criterion (a specific indicator of the *FC* level of cracking of the surface of this pavement). The criterion used in determination of *N*_f(lab)_ concerns a 50% decrease in the stiffness modulus of the mixture of the lowest asphalt layer at the level of cyclic strain amplitude of this mixture equal to *ε*_h_. However, they are useful for comparisons with fatigue life values *N*_f(SiM)_ (obtained from the proposed Similarity Method).

## 4. Calculation Results and Discussion

In Table 6, the coefficients *A* and α from formula (1) for the three methods of determination of the design lifespan of the RS and HB pavement structures (varying in binders used in the AC 22 asphalt base course) are listed. For the AASHTO 2004 method the cracking index *FC* = 5% was assumed.

Critical strain occurs directly in all three formulas, raised to the power equal to the coefficient *α*, whose values are 3.9 and greater. This implies that life prediction is very sensitive to changes in critical strain values.

The coefficient *α*, which indicates the slope of the fatigue curve plotted on a logarithmic scale, has the same value for all structures in the “structural fatigue life” formulas regardless of the presence of HiMA binder. In the case of “laboratory fatigue life” formulas, the values of this coefficient vary depending on the type of asphalt mixture used and are significantly higher than those in “structural fatigue life” formulas. Therefore, in the proposed SiM formulas, the *α* values for the structure with HiMA binder in the asphalt base course are higher than for RS structures.

The *A* coefficient in the “structural fatigue life” formula, although it depends on the physical properties of the material, has a similar value for both structure variants. This does not reflect the increased resistance of HiMA mixtures to repetitive strain, which is clearly visible in laboratory formula, where the *A* coefficient for these mixtures has significantly higher values. In the case of “SiM fatigue life” formula, the *A* coefficient value is identical to this of the “structural fatigue life” formula for the reference structure and different (greater) for HiMA structures (but smaller than for “laboratory fatigue life” formula).

In summary, since two different asphalt mixtures used in the base courses of the analyzed pavements were investigated, two different fatigue curves were obtained based on laboratory tests. Moreover, the “structural” fatigue curves—parallel, according to the AASHTO formulas—and two fatigue curves from “SiM” were obtained as well.

**Figure 2:**
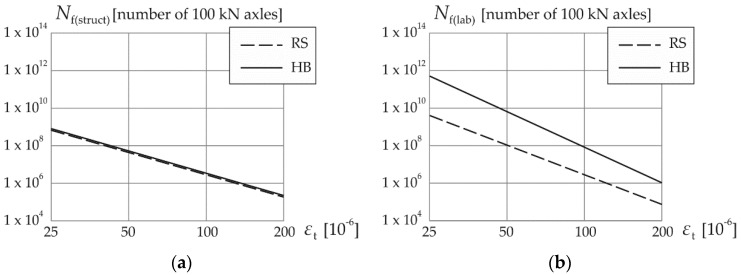
Fatigue curves of the entire structures with asphalt base course fatigue characteristics based on formula (1) and: (**a**) structural method at *FC* = 5%, (**b**) laboratory method.

**Figure 3:**
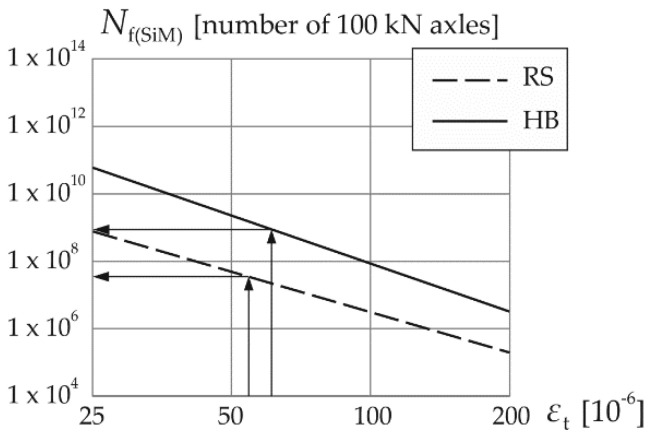
Fatigue curves of the entire structures with asphalt base course fatigue characteristics based on formula (1) and the SiM method; the resulting predictions of the pavement life for the critical strain values presented in Table 7 are marked.

Figure 2 and Figure 3 show fatigue curves (straight lines on a log-log plot) of pavement structures depending on the fatigue characteristics of mixtures used in the base course. The curves were obtained based on formula (1), using the SiM method as well as the “laboratory” and “structural” methods of life prediction due to fatigue “bottom-up” cracking. Figure 3 also shows the method of determining the life of the pavement for a given value of critical strain *ε*_t_.

The “laboratory” fatigue curves presented in Figure 2b show significantly higher fatigue resistance of the mix with HiMA binder compared to the standard mix (unmodified). However, in Figure 2a it is visible that the fatigue curves determined from the AASHTO formula do not reflect this property of the asphalt mix with HiMA—fatigue curves of both mixtures are very close to each other. These formulas correctly determine the fatigue life for unmodified asphalt structures only. Fatigue curves according to the proposed SiM method shown in Figure 3 overlap with the AASHTO curves for the standard mixes, while for the highly modified mixes they do take into account the differences in fatigue resistance.

Table 7 presents a comparison the critical strains and the corresponding design life values determined using the SiM method as well as the “laboratory” and “structural” methods described in Section 3.

The fatigue lifespans obtained according to the AASHTO 2004 method should not be compared directly (in terms of value) with the results of the fatigue lifespans according to the laboratory curves of the material used in the asphalt base course. The defining criteria of the methods *N*_f(struct)_ and *N*_f(lab)_ are disproportionate, as may be observed in their values for the base course asphalt mix with road binder and, especially, with highly modified binder HiMA. Therefore, the *N*_f(SiM)_ fatigue life is thought to be a realistic adjustment of the *N*_f(struct)_ value, taking into account the different properties of mixtures in the base course and showing an increase in the life on the basis of fatigue equations. On the other hand, *N*_f(SiM)_ fatigue life is intended as an adjustment of the *N*_f(lab)_ value as well, taking into account the structure of the pavement and including the criterion of cracking index *FC* on the basis of the AASHTO 2004 equations.

In terms of absolute values, the obtained lifespan predictions for pavements with highly modified binders may seem debatable, but in comparative terms, in relation to reference structures, they seem justified and interesting.

The predicted fatigue life of HiMA pavements according to the SiM formulas is significantly greater than the values calculated according to the AASHTO formulas, but it is also in the appropriate qualitative relation to the “laboratory” life (rounded to whole number values, it is two to three times smaller). The design lifespan according to the SiM formula for HB structure increases about 20 times compared to RS structure. It is noteworthy that this number is similar to the number estimated in previous studies [6], where the value of 64 was assumed.

## 5. Method Testing

The Similarity Method was subsequently applied in several variants of calculations, testing its reaction to variability in basic parameters of the pavement considered in Section 4. The following factors were assumed to be variable:

- gradation of the asphalt mixture used in the base course, changing from AC 22 to AC 16 with a higher binder content (material sensitivity)—HB-A structure (mix AC 16 W 45/80-80 HiMA in the HB-A structure and AC 16 W 35/50 in its reference structure, material data not previously mentioned are shown in Table 8),

- thickness of the AC 22 PMB 45/80-80 HiMA asphalt base (geometric sensitivity), change from 12 cm to 16 cm—HB-B structure,
- strengthening of the improved subgrade layer (sensitivity due to subgrade stiffness), change from 300 MPa to 400 MPa—HB-C structure,
- value of the assumed *FC* index of bottom-up fatigue cracks on the road surface, change from 5% to 10% (sensitivity due to the definition of fatigue failure of the pavement)—HB-D structure.

Table 9 presents a comparison of the results of fatigue life calculations for the above pavement cases.

In order to compare the sensitivity of the Similarity Method in relation to the results of the AASHTO 2004 method, the ratio HB-X/HB, representing the change in lifespan of the HB-X structure in relation to the results obtained for the HB structure, was introduced. As visible in Table 9, the ratio HB-X/HB reaches similar values for the SiM method and AASHTO 2004 method with the exception of the structure HB-A (change of mixture type from AC 22 to AC 16 in the asphalt base course). It can therefore be assumed that the SiM method is most sensitive to the properties of the material used in the asphalt base course, including its characteristics such as air voids content and effective binder volume. For the remaining three variables, the expected change trends were obtained.

## 6. Conclusions

1. The Similarity Method is promising as means of rough estimation of fatigue life under structural conditions and avoids the problems associated with classical calculation methods of fatigue life, e.g., the Asphalt Institute method and AASHTO 2004. It can be assessed that the Similarity Method enables taking into account the predicted “fatigue life of the asphalt mixture under laboratory conditions” in estimations of the “structural fatigue life” for new asphalt materials with different properties than those assumed in the AI, AASHTO and similar equations, e.g., when a highly modified binder (HiMA) is used in the lowest asphalt layer.
2. The “SiM” hypothesis proposed in this paper assumes that the lifespan values obtained using the AASHTO method are realistic (on the basis of a certain projection) and to some extent proportional to the relation between laboratory fatigue characteristics of the mixtures with and without HiMA used in the asphalt base courses. It proved suitable for evaluation of the fatigue life of asphalt mixtures with HiMA. This will enable inclusion of new materials, such as HiMA asphalt mixtures, in pavement structure design.
3. Sensitivity analysis of the SiM method with four different variables (material, geometric, subgrade, cracking index) showed that the SiM method is sufficiently sensitive to changes in these variables. It was also noted that the greatest sensitivity of the method is exhibited in the case of material used in the asphalt base course (change from AC 22 to AC 16)—increasing *N*_f(SIM)_ about 12 times. The change in the *FC* crack rate from 5% to 10% resulted in a change (increase) in *N*_f(SiM)_ over 2 times. This is similar to the effect of increasing the thickness of the asphalt base by 33% (from 12 to 16 cm). The impact of the improved subgrade strengthening turned out to be the smallest (a 20% change in *N*_f(SiM)_) among the tested parameters.
4. Based on the results of *N*_f(SiM)_ tests, obtained indirectly from laboratory fatigue tests of asphalt mixtures, it was possible to rank the tested structures according to the influence of the binder and the type of mixture on the service life of the structure *N*_f_ due to fatigue cracking. The obtained ranking according to *N*_f(SIM)_ was in accordance with the current state of knowledge on the behavior of different types of binder and asphalt. Therefore, in comparison with classical calculation methods, the correct result of assessment of the tested structures was obtained.
5. The SiM method is relatively simple, and the associated efforts are limited to sample preparation and testing in order to determine the laboratory fatigue equation. The tests are performed on samples of the asphalt base course of the analyzed pavement and its reference pavement with standard neat binders. This effort is significantly lower than the effort of construction of a pavement test section and determination of its effective fatigue life through testing.

## Figures and Tables

**Table 1:** Basic properties of the binders used in the asphalt mixtures.

Binder	Specification	Penetration@25 °C EN 1426 (0.1 mm)	Softening Point R&B EN 1427 (°C)	Elastic Recovery EN 13398 (%)	PG AASHTO M320
35/50	EN 12591	43	53.5	-	70–22
PMB 45/80-80 HiMA	EN 14023 Polish appendix 2014	69	92	95	88–28
PMB 45/80-55	EN 14023 Polish appendix 2014	65	59	85	70–28

**Table 2:** Material parameters of asphalt mixtures for the Huet-Sayegh model of asphalt layers at T = 10 °C.

Asphalt Mixture	*η*_a_ (MPa·s)	*η*_b_ (MPa·s)	*E*_a_ (MPa)	*E*_p_ (MPa)	*k*_a_ (-)	*h*_b_ (-)
SMA 8 PMB 45/80-55	1053	2790	28,500	200	0.22	0.66
AC 16 35/50	19,482	66,240	27,600	210	0.26	0.73
AC 22 35/50	36,717	110,152	18,800	450	0.26	0.80
AC 22 PMB 45/80-80 HiMA	4463	16,065	18,900	500	0.27	0.73

**Table 3:** Material parameters of asphalt mixtures (cont.).

Asphalt Mixture	Dynamic Modulus T = 10 °C, f = 10 Hz |E*|(MPa)	Poisson’s Ratio *ν* (-)	Binder Content *V*_a_ (% *v*/*v*)	Voids in Asphalt Mix *V*_v_ (% *v*/*v*)
SMA 8 PMB 45/80-55	8087	0.3	16.36	2.2
AC 16 35/50	14,818	0.3	10.49	5.4
AC 22 35/50	11,563	0.3	9.86	5.2
AC 22 PMB 45/80-80 HiMA	8668	0.3	9.93	5.8

**Table 4:** Material parameters of lower pavement layers.

Layer (Material)	Young’s Modulus of Elasticity *E* (MPa)	Poisson’s Ratio *ν* (-)
Subbase (mechanically stabilized aggregate)	400	0.30
Improved subgrade (soil stabilized with cement)	300	0.30
Natural subgrade (natural soil)	100	0.35

**Table 5:** Fatigue coefficients of the tested asphalt mixtures according to formula (10).

Asphalt Mixture for Asphalt Base Course	*A*_1_	*A*_0_
AC 22 35/50	−5.2853	17.007
AC 22 PMB 45/80-80 HiMA	−6.3487	20.598

**Table 6:** Values of *A* and *α* coefficients from the formula (1) (fatigue curves) for the three methods of determination of the design lifespan of the structure at *FC* = 5%.

Method	Coefficient	Pavement Structure Variant	
		RS	HB
AASHTO 2004 equations	*A*_struct_	14.386	14.433
	*α*_struct_	3.9492	3.9492
Laboratory	*A*_lab_	17.007	20.598
	*α*_lab_	5.2853	6.3487
SiM	*A*_SiM_	14.386	17.424
	*α*_SiM_	3.9492	4.7438

**Table 7:** Design life of the pavement structures analyzed in the work, determined using formula (1) and coefficients given in Table 6.

Critical Strains and Predicted Fatigue Life [Millions of 100 kN Axles]	Pavement Structure Variant	
	RS	HB
ε_h_ (10^−6^)	54.1	63.1
*N*_f(struct)_	35	21
*N*_f(lab)_	70	1474
*N*_f(SiM)_	35	765

**Table 8:** Huet-Sayegh model parameters, Poisson’s ratio and AC 16 W 45/80-80 HiMA fatigue curve coefficients at *T* = 10 °C.

*η*_a_ (MPa·s)	*η*_b_ (MPa·s)	*E*_a_ (MPa)	*E*_p_ (MPa)	*k*_a_ (-)	*h*_b_ (-)	*ν* (-)	*A*_1_	*A*_0_
3019	9662	27,500	230	0.26	0.58	0.3	−7.0748	22.698

**Table 9:** Results of application of the SiM method and the AASHTO method (as a reference) for selected modifications of the HB pavement structure.

Critical Strains, Coefficients and Predicted Fatigue Life		Pavement Structure Variant				
		HB	HB-A	HB-B	HB-C	HB-D
	ε_h_ (10^−6^)	63.1	57.3	51.2	61.0	63.1
AASHTO 2004 equations	*A*_struct_	14.433	14.407	14.425	14.433	14.737
	*α*_struct_	3.9492	3.9492	3.9492	3.9492	3.9492
	*N*_f(struct)_ [millions of 100 kN axles]	21	29	47	24	42
	HB-X/HB ratio	1.0	1.4	2.2	1.1	2.0
SiM	*A*_SiM_	17.424	20.349	17.414	17.424	17.792
	*α*_SiM_	4.7438	5.8960	4.7438	4.7438	4.7438
	*N*_f(SiM)_ [millions of 100 kN axles]	765	9661	2031	903	1786
	HB-X/HB ratio	1.0	12.6	2.7	1.2	2.3

## Data Availability

Publicly available datasets were analyzed in this study. This data can be found in the book [19]: https://www.orbiton.pl/ (accessed on 18 January 2021).
